# Deletion of the lipid droplet protein kinase gene affects lipid droplets biogenesis, parasite infectivity, and resistance to trivalent antimony in *Leishmania infantum*

**DOI:** 10.1371/journal.pntd.0011880

**Published:** 2024-01-18

**Authors:** Juliana Martins Ribeiro, Paula Alves Silva, Héllida Marina Costa-Silva, Ana Maria Murta Santi, Silvane Maria Fonseca Murta

**Affiliations:** Grupo Genômica Funcional de Parasitos, Instituto René Rachou, Fiocruz Minas, Belo Horizonte, Minas Gerais, Brazil; Institut national de la recherche scientifique, CANADA

## Abstract

The Lipid Droplet Protein Kinase (LDK) facilitates lipid droplet (LD) biogenesis, organelles involved in various metabolic and signaling functions in trypanosomatids. As LDK’s function has not been previously explored in *Leishmania spp*., we utilized CRISPR/Cas9 technology to generate *LDK*-knockout lines of *Leishmania infantum* to investigate its role in this parasite. Our findings demonstrate that *LDK* is not an essential gene for *L*. *infantum*, as its deletion did not impede parasite survival. Furthermore, removing *LDK* did not impact the growth of promastigote forms of *L*. *infantum* lacking *LDK*. However, a noticeable reduction in LDs occurred during the stationary phase of parasite growth following LDK deletion. In the presence of myriocin, a LD inducer, *LDK*-knockout parasites displayed reduced LD abundance during both logarithmic and stationary growth phases compared to control parasites. Moreover, an infection analysis involving THP-1 cells revealed that 72 h post-infection, *LDK*-knockout *L*. *infantum* lines exhibited fewer infected macrophages and intracellular amastigotes than control parasites. *LDK*-knockout *L*. *infantum* lines also displayed 1.7 to 1.8 -fold greater resistance to trivalent antimony than control parasites. There were no observed alterations in susceptibility to amphotericin B, miltefosine, or menadione in *LDK*-knockout *L*. *infantum* lines. Our results suggest that LDK plays a crucial role in the biogenesis and/or maintenance of LDs in *L*. *infantum*, as well as in parasite infectivity and resistance to trivalent antimony.

## Author summary

Lipid droplets (LDs) are organelles with diverse cell metabolism and signaling roles. Lipid droplet protein kinase (LDK) is responsible for LD formation in trypanosomatids, but its function in *Leishmania* spp. remains unknown. In this study, we demonstrate that deleting the *LDK* gene in *Leishmania infantum* has no impact on parasite growth. However, it reduces the amount of LDs during the stationary phase of parasite growth. When exposed to myriocin, known to induce LD production, we observed a decrease in LDs during both the logarithmic and stationary phases of *LDK*-knockout parasites. Additionally, *LDK*-deficient parasites exhibit reduced infectivity and increased resistance to trivalent antimony. In summary, our findings suggest that LDK plays a crucial role in the formation and/or maintenance of LDs in *L*. *infantum*.

## Introduction

Leishmaniasis encompasses a complex of diseases caused by over 21 distinct species of protozoan parasites belonging to the genus *Leishmania*. It is transmitted to humans through the bites of infected female sand flies [1]. This disease predominantly affects vulnerable populations across more than 98 countries, spanning tropical and subtropical regions in the Americas, Africa, Asia, Europe, and Oceania. Over one billion people live at risk of infection in these endemic areas, resulting in approximately 0.7 to 1 million new cases annually [1,2].

Leishmaniasis manifests in two primary clinical forms: cutaneous leishmaniasis (CL), characterized by cutaneous and/or mucosal lesions, and visceral leishmaniasis (VL) [3]. VL, caused by *Leishmania* (*Leishmania*) *donovani* in Asia and Africa and *Leishmania* (*Leishmania*) *infantum* in the Mediterranean Basin, the Middle East, Central Asia, South America, and Central America, represents the most severe form of the disease and can be fatal without prompt treatment [3,4]. The progression of infection and clinical manifestations of leishmaniasis are influenced by both parasite-related factors (species, strain, life stage, initial parasitic load) and host-related factors (immune and nutritional status, age, presence of co-morbidities) [2–4]. Currently, no vaccines are available, and the absence of effective vector control programs underscores the reliance on chemotherapy as the primary strategy for managing all forms of leishmaniasis. The drugs used for leishmaniasis treatment include pentavalent antimonials, amphotericin B formulations, and miltefosine. However, the emergence of drug-resistant parasite strains and undesirable drug side effects contribute to treatment failures [5].

Given this context, developing novel therapeutic approaches against leishmaniasis is paramount, with the lipid droplet protein kinase (LDK) emerging as a promising molecular target. Initial investigations into LDK in *Trypanosoma brucei* revealed its involvement in lipid droplet (LD) formation and biogenesis [6]. LDs, also referred to as lipid bodies, adiposomes, or oil bodies in plants, are cytoplasmic organelles composed of a hydrophobic core containing triglycerides and cholesteryl esters enveloped in a phospholipid monolayer, cholesterol, and a set of associated proteins [7–9]. LDs serve various roles in cellular metabolism, homeostasis, and signaling [7–9] and are present in virtually all cell types and organisms, including protozoan parasites [10].

The mechanisms governing LD biogenesis in trypanosomatids remain incompletely elucidated and may involve multiple proteins [11]. To date, only LDK [6] and Lipin (TbLpn) [12] have been identified as enzymes participating in LD formation in *T*. *brucei*. In mammalian cells, the PAT protein family, which includes Perilipin, Adipose differentiation-related protein (ADRP), and Tail-interacting protein 47 kDa (TIP47), plays a structural role in LD formation [11,13]. Genes related to PAT proteins have not yet been identified in *Leishmania* spp. genomes, making LDK the only known protein with a comparable function [11].

LDK is primarily located at the periphery of LDs [6,14], and it possesses all the motifs and residues necessary for protein kinase activity, followed by a 24-amino acid transmembrane domain similar to *T*. *brucei* [6]. The kinase activity of LDK is mediated through autophosphorylation. Moreover, studies have demonstrated that *T*. *brucei* mutant parasites with reduced *LDK* expression, generated via RNA interference, displayed minimal growth defects but exhibited a 90% reduction in LD formation [6]. LDK localization was assessed in *Leishmania mexicana* using CRISPR/Cas9 gene editing, employing labeling with the fluorescent protein mNeonGreen [14]. As observed in *T*. *brucei*, LDK in *L*. *mexicana* is also found in LDs. Nevertheless, the specific function of LDK in *Leishmania* spp. remains unknown.

To unravel the role of LDK in *L*. infantum, we employed the CRISPR/Cas9 approach to disrupt the *LDK* gene. We assessed the phenotypes of these mutant parasites in terms of growth rate, LD production, infectivity in THP-1 macrophages, and susceptibility to trivalent antimony (Sb^III^), amphotericin B, miltefosine, and menadione.

## Methods

### Parasites culture conditions

*Leishmania (Leishmania) infantum* RPV (MHOM/BR/2002/LPC-RPV) promastigotes were cultured at 26°C in M199 medium (Gibco, Waltham, MA, USA) supplemented with the following components: 40 mM HEPES (pH 7.4), 5 μg/mL hemin, 2 μg/mL biopterin, 1 μg/mL biotin, 2 mM L-glutamine, 500 U/mL penicillin, 50 μg/mL streptomycin, and 10% inactivated fetal bovine serum. To maintain the cultures, we conducted biweekly passages, inoculating 1 × 10^6^ parasites for each 5 mL of medium. Unless specified otherwise, all experiments were conducted using promastigotes in the logarithmic growth phase.

### Generation of *LDK L*. *infantum* knockout

To generate *LDK* (LINF_280026600) null mutants, we employed the CRISPR/Cas9 method, following the procedure outlined in a previous study [15]. Plasmid pTB007, containing hygromycin as a resistance marker, was utilized to express SpCas9 and T7RNAP. Parasites carrying this plasmid, successfully expressing Cas9, were subjected to transfection with donor DNAs and guide RNA templates chosen using the LeishGEdit tool. The sgRNA templates were generated through polymerase chain reaction (PCR) employing G00 primer. Donor DNAs aimed at deleting the *LDK* gene were amplified via PCR, using specific primer pairs along with plasmids pTNEO_v1 and pTBlast_v1 as templates. These plasmids contain sequences that confer resistance to the antibiotics neomycin (NEO) and blasticidin (BLAST), respectively. Promastigote forms were transfected [16] with equimolar amounts of sgRNA and donor DNAs, as previously described [15]. Subsequently, parasites were selected using 40 μg/mL of NEO and 30 μg/mL of BLAST. Clones were further selected in semi-solid M199 medium with the addition of selective drugs. However, it’s important to note that all experiments aimed at determining parasite phenotypes were conducted without the use of selection drugs. All primers used to construct the plasmids and DNA fragments are listed in S1 Table.

### Add-back parasites

The *LDK* coding sequence was cloned into the pIR1_SAT vector to generate the add-back parasites. After ligation, the resulting plasmids were incubated with *Escherichia coli* TOP10F’ (Invitrogen, Waltham, MA, USA) at 4°C for 30 minutes and then at 42°C for 45 seconds using a dry bath. Following this, the bacteria were incubated at 37°C for 1 hour to induce expression of the ampicillin resistance gene and subsequently plated on solid LB medium containing ampicillin (Sigma, St. Louis, MO, USA). Clones possessing the correct LDK sequence, confirmed via colony PCR, were subjected to digestion with the restriction enzymes *Bgl*II (BioLabs, Durham, NC, USA), *Sac*II (Promega, Madison, WI, USA), and *Eco*RV (Promega) to verify the orientation of the gene within the plasmid. Subsequently, 100 μg of the pIR1_SAT_LDK plasmid was linearized with using the restriction enzyme *Swa*I (BioLabs) and precipitated using sodium acetate and isopropanol. Transfection of ΔLDK mutant promastigotes with the pIR1_SAT_LDK plasmid was conducted following the established protocol [16], and the parasites were selected using 100 μg/mL of nourseothricin sulfate (Sigma).

### PCR reactions

To screen for clones and add-back parasites, we extracted genomic DNA from the parasites using the phenol/chloroform method. The complete *LDK* coding sequence was then amplified via PCR, and the PCR product was analyzed using agarose gel electrophoresis. To evaluate *LDK* transcript levels, we extracted total RNA using the TRIzol (Invitrogen)/chloroform method. Subsequently, total RNA was treated with DNase I (Ambion, Waltham, MA, USA), and cDNA synthesis was carried out using Superscript II reverse transcriptase (Invitrogen) following the manufacturer’s instructions.

To quantify the number of copies and the transcript levels of the LDK gene, we diluted all genomic DNA and cDNA samples to 100 ng/μL and used them in the RT-qPCR amplification reaction. The amplification was performed with 1X SYBR GREEN master mix (Applied Biosystems, Waltham, MA, USA) and the specific primers listed in S1 Table. DNA polymerase I served as a normalizer and an endogenous control gene. All reactions were conducted in triplicate in a 96-well plate. The amplification process involved an initial cycle of 50°C for 2 minutes and 95°C for 10 minutes, followed by 40 cycles of 95°C for 15 seconds and 60°C for 1 minute each, and concluded with a final cycle of 95°C for 15 seconds, 60°C for 15 seconds, and 95°C for 15 seconds. Relative expression levels were calculated using the 2^-ΔΔCT^ method [17], normalized to the constitutive gene DNA polymerase I. We performed three independent biological replicates for each parasite line, with each replicate conducted in triplicate.

### Growth curve of promastigotes forms

*L*. *infantum* promastigotes (1 × 10^6^ parasites/mL) were maintained in M199 medium at 26°C, and the number of parasites was determined daily using theZ1 Coulter Particle Counter (Beckman Coulter, Brea, CA, USA). Three independent experiments were performed in triplicates.

### Nile red and Bodipy 493/503 staining and quantification

*L*. *infantum* promastigotes (3 × 10^6^ parasites) in both the logarithmic and stationary growth phases were cultured for 24 h, with or without 1.5 μM myriocin (Sigma-Aldrich) [6,18]. The parasites were then washed twice with phosphate-buffered saline and stained with Nile red (1.5 μg/mL) (Sigma-Aldrich) or Bodipy 493/503 (5 μg/mL) (Thermo Fisher Scientific) for 30 min at 25°C under dark. To assess the quantity of lipid droplets within the parasites, we acquired a total of 30,000 events/sample using the BD FACSCalibur flow cytometer (BD Biosciences, Franklin Lakes, NJ, USA), and the data were analyzed using Flow Jo software (Flow Cytometry Analysis Software, version 10) [19]. For confocal microscopy, a portion of each cell suspension was incubated on a glass coverslip pre-coated with 0.1% poly-L-lysine for 10 min and subsequently fixed in 4% formaldehyde for an additional 10 min at 25°C, in dark. The glass coverslips were washed thrice with phosphate-buffered saline and incubated with ProLong Gold antifade reagent. We captured images using a Zeiss LSM 880 confocal microscope (Oberkochen, Germany) with a 63x objective and numerical aperture of 1.4. The acquired images were analyzed using the Zeiss Zen 3.8 microscopy software. Five independent experiments were performed in duplicate.

### THP-1 derived macrophages infection

THP-1 cells (human monocytes lineage) were cultured in RPMI-1640 medium supplemented with 2 mM glutamine, 100 U/mL penicillin and 100 μg/mL streptomycin antibiotics, and 10% fetal bovine serum, and maintained at 37°C and 5% CO_2_. For facilitating the differentiation of monocytes into macrophages, the cells were incubated with 20 ng/mL of phorbol myristate acetate in 24-well plates on glass coverslips, at 37°C and 5% CO_2_. After 72 h, the macrophages were infected with *Leishmania* cultures on the second day of the stationary phase (10 parasites per macrophage) for 3 h. The parasites that failed to infect the macrophages were washed away, and the infected macrophages were incubated for 72 h in RPMI-1640 medium. The infectivity of *L*. *infantum* mutants and add-backs was evaluated immediately after the 3 h incubation period as well as after 72 h. Slides were stained with Rapid Panoptic (Laborclin, Pinhais, PR, Brazil), photographed, and infection was assessed by counting the number of infected macrophages and intracellular amastigotes using the ImageJ software (National Institutes of Health, Bethesda, MD, USA). Three independent experiments were performed in duplicate.

### Effective concentration (EC_50_) assays

The susceptibility of the parasites to different drugs was assessed using 2 × 10^5^ *L*. *infantum* promastigotes/mL incubated in 1 mL of M199 medium containing different drug concentrations ranging from 50 to 250 μM of Sb^III^, 0.05 to 0.6 μM of amphotericin B, 5.0 to 40 μM of miltefosine, and 1.0 to 8 μM of menadione. After 48 h of incubation, the number of parasites grown in the absence and presence of each drug was determined by using the Z1 Coulter Particle Counter (Beckman Coulter). The values of the effective concentration necessary to reduce growth by 50% (EC_50_) were obtained from three independent experiments in triplicate, using the linear interpolation method [20].

### Statistical analysis

Data were analyzed using GraphPad Prism v.8.2.0 software (San Diego, CA). Ordinary one-way ANOVA or two-way ANOVA test with Dunnett’s post-test were used to compare Cas9 and mutant parasites. Data are presented as mean ± standard error of the mean. Statistically significant differences were considered for *p* lower than 0.05.

## Results

### Generation and characterization of *LDK* null mutants

**Fig 1:**
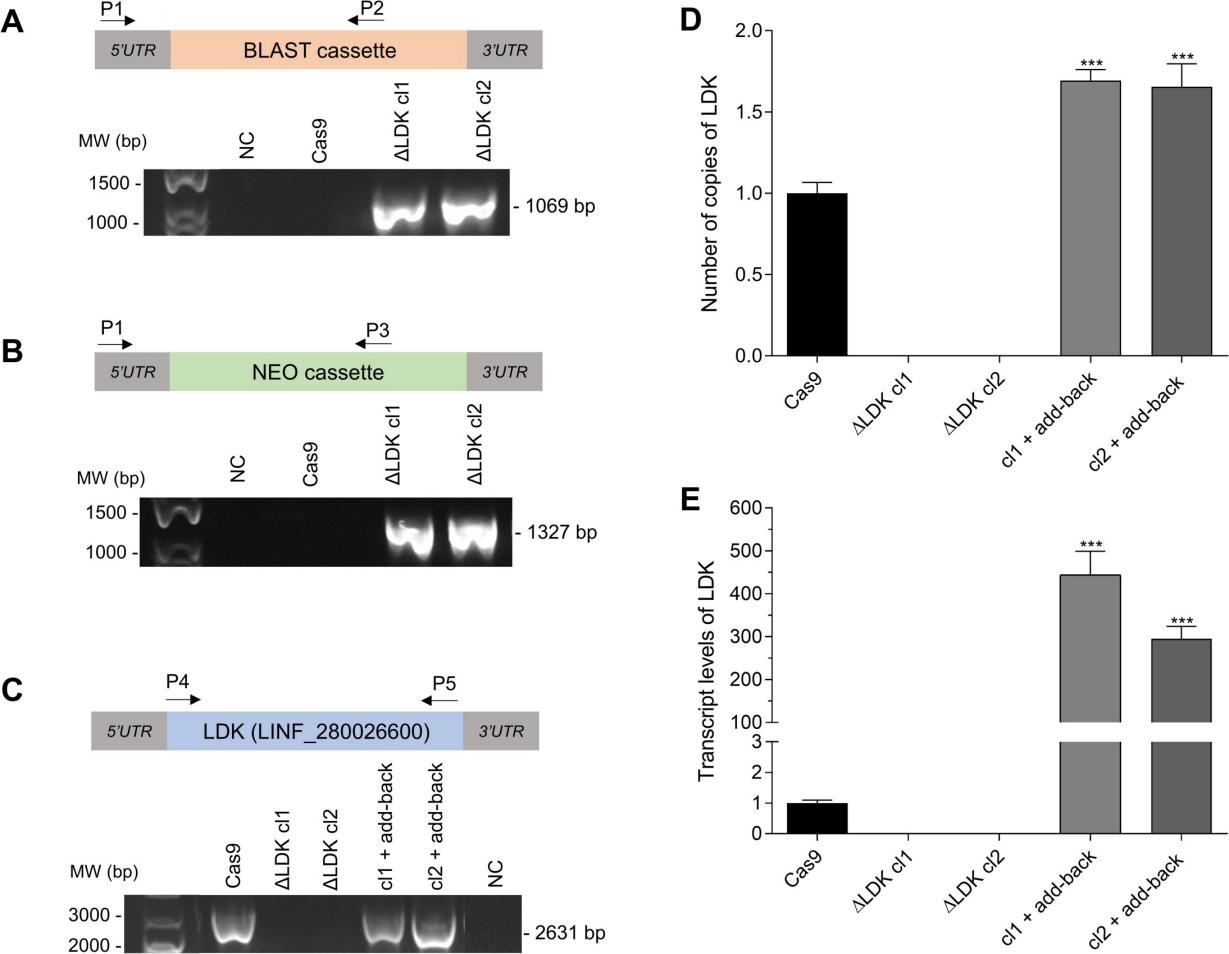
Characterization of *Leishmania infantum* ΔLDK knockout clones generated using CRISPR/Cas9 and (LDK) add-back parasites. (A) The correct integration of the resistance markers BLAST (blasticidin) (1069 bp) and (B) NEO (neomycin) (1327 bp) was evaluated using PCR by annealing a primer in a 3′UTR region adjacent to the cassette (primer P1) and another primer annealed within each resistance marker sequence (primers P2 or P3). (C) The complete *LDK* coding sequence (2631 bp) was amplified using PCR with primers P4 and P5. RT-qPCR was performed with specific primers and the number of copies of the *LDK* gene were analyzed using the 2^-ΔΔCT^ method and normalized to the constitutive gene DNA polymerase I. (D) Number of copies of the *LDK* gene. (E) Transcript levels of *LDK*. One-way analysis of variance (ANOVA) with Dunnett’s post hoc test was used to compare control parasite (Cas9) and add-back parasites. *represents significant differences in relation to the control parasite (Cas9) (*** *p* < 0.001). MW: molecular weight; NC: negative control; bp: base pair.

The genome of the JPCM5 strain of *L*. *infantum* harbors a single copy of the *LDK* gene (TritrypDB accession number LINF_280026600), located on chromosome 28. This gene spans 2631 base pairs and encodes a protein comprising 876 amino acids. To generate *LDK* knockout cell lines, we employed the CRISPR/Cas9 methodology, following established protocols [15]. Confirmation of the correct integration of both the BLAST and neomycin cassettes into the *L*. *infantum* genome was achieved through PCR analysis in all tested mutant *L*. *infantum* clones (Fig 1A and 1B). Additionally, PCR was employed to verify the deletion of *LDK* in these parasites, successfully establishing *LDK*-null mutants. These generated clones were designated as ΔLDK cl1 and ΔLDK cl2 (Fig 1C). The reintroduction of the *LDK* gene was confirmed via PCR, and the resulting add-back parasites were labeled as cl1 + add-back and cl2 + add-back (Fig 1C). As depicted in Fig 1D, the number of *LDK* gene copies indicate complete deletion in the knockout clones, which was subsequently reinstated in the add-back parasites. Fig 1E illustrates the transcript levels of the *LDK* gene; in *LDK*-knockout parasites, *LDK* expression was absent, while the add-back parasites exhibited a restored genotype.

### LDK is not essential for *L*. *infantum* survival, and its deletion did not alter parasite growth

The growth of the promastigote forms of *L*. *infantum* parasites expressing Cas9 and ΔLDK mutant clones cl1 and cl2 was determined by counting the parasites every 24 h. No difference in growth was observed among parasites expressing Cas9, ΔLDK mutant parasites, as well the add-back parasites (S1 Fig). The results demonstrated that *LDK* is not an essential gene for *L*. *infantum*, and its deletion did not alter parasite growth.

### *LDK* knockout reduced LD production in the stationary phase

**Fig 2:**
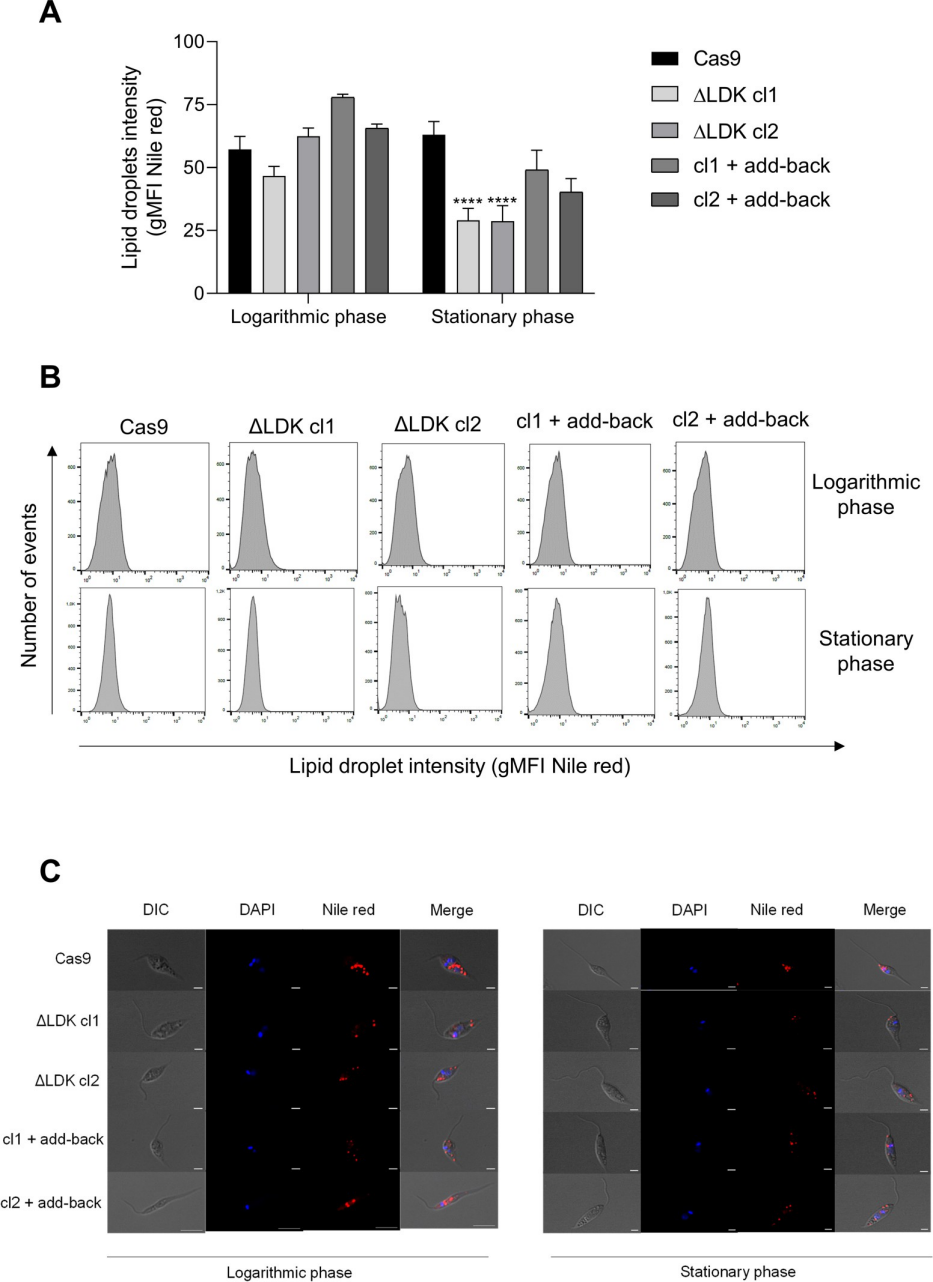
Quantification of lipid droplets of Cas9, *LDK*-knockouts, and add-back parasites. (A) The graphs represent the mean geometric fluorescence intensity (gMFI) of Nile red. (B) Representative histograms of gMFI of the Nile red. (C) Representative images of lipid droplets obtained using confocal microscopy. Two-way ANOVA with Dunnett’s post hoc test was used to compare Cas9-expressing controls and *LDK*-knockouts for each growth phase. *represents significant differences between Cas9 and ΔLDK cl1 and ΔLDK cl2 knockouts (**** *p* < 0.0001). Bars: 10 μm.

To evaluate the effect of *LDK* deletion on the formation of LDs in *L*. *infantum*, we quantified LDs using flow cytometry based on the geometric mean fluorescence intensity (gMFI) of Nile red (Fig 2) and Bodipy 493/503 (S2 Fig) staining in the logarithmic and stationary phases of parasite growth. No significant difference was observed in the amount of LDs in the knockout parasites in the logarithmic phase when compared to that of the Cas9-expressing controls, and the profile did not change with the reintroduction of the *LDK* gene into the add-back parasites (Figs 2A and S2A). However, in the stationary phase, a reduction in the amount of LDs in *LDK*-knockout parasites (Figs 2A and S2A) was observed. Reintroduction of the *LDK* gene into knockout clones recovered the phenotype. The gMFI representative histograms are shown in Figs 2B and S2B. Representative images of LDs obtained using confocal microscopy are shown in Figs 2C and S2C.

### *LDK* deletion affected biogenesis and maintenance of LDs in *L*. *infantum*

**Fig 3:**
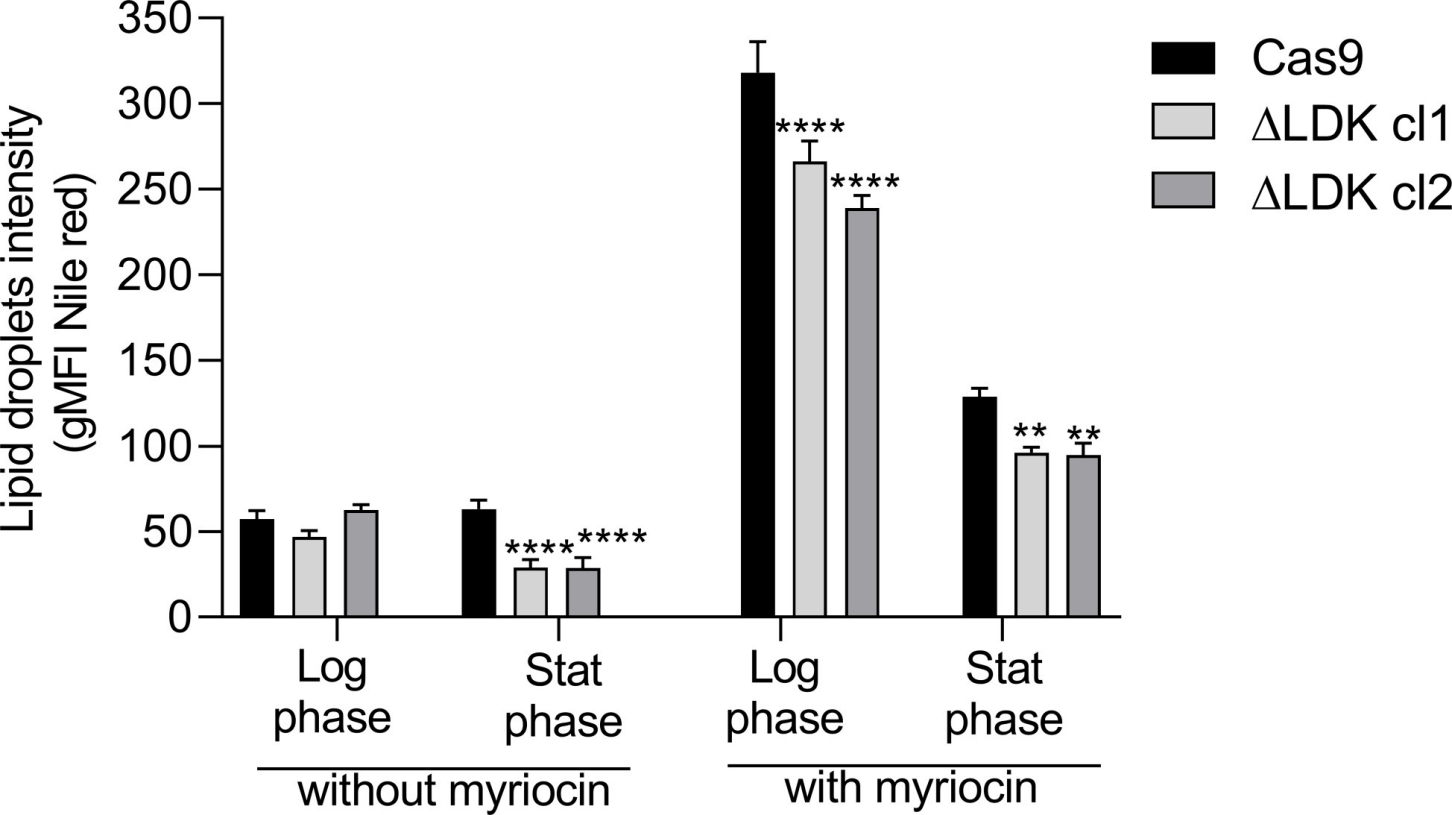
Quantification of lipid droplets in the absence and presence of myriocin. The graph represents the gMFI of Nile red-labeled parasites. One-way ANOVA with Dunnett’s post hoc test was used to compare Cas9-expressing controls and *LDK*-knockouts. *represents significant differences in relation to the Cas9-expressing controls (** *p* < 0.01 and **** *p* < 0.0001). Log phase, Logarithmic phase; Stat phase, Stationary phase.

Because our results indicated that *LDK* deletion leads to a reduction in LD production during the stationary growth phase of *L*. *infantum*, we aimed to investigate how *LDK*-knockout parasites respond in the presence of a substance that stimulates LD production. To achieve this, we incubated both the Cas9-expressing control and mutant parasites with 1.5 μM myriocin, a specific inhibitor of the serine palmitoyltransferase enzyme and an inducer of LD production [18], and then assessed LD production. This concentration has previously been utilized in assays involving *T*. *brucei* [6,18]. Notably, the 1.5 μM myriocin treatment did not result in a reduced growth rate or cell viability for any of the parasites during the evaluated period. Furthermore, myriocin treatment increased LD production in both the Cas9-expressing controls and *LDK*-knockout clones during both growth phases, compared to the untreated conditions (Fig 3). In the logarithmic growth phase, the geometric mean fluorescence intensity (gMFI) of Nile red-stained LDs was 57.2 and 318.1 for Cas9-expressing controls, and 54.6 and 252.6 for ΔLDK cl1/cl2 parasites, in the presence and absence of myriocin, respectively. In the stationary phase, the gMFI of Nile red-stained LDs was 63.0 and 128.8 for Cas9-expressing controls, and 28.9 and 92.3 for ΔLDK cl1/cl2 parasites, in the presence and absence of myriocin, respectively. A significant reduction in the amount of LDs was observed in *LDK*-knockout clones during both growth phases when compared to the Cas9-expressing controls in the presence of myriocin (Fig 3). This reduction in LDs in the stationary growth phase of *LDK*-knockout clones was consistent whether myriocin was present or absent (Fig 3). This observation is noteworthy, as without myriocin, *LDK*-knockout clones exhibited a reduction in LD abundance only during the stationary phase (Figs 2A and 3 and S2A).

### *LDK* deletion altered the intracellular proliferation profile

**Fig 4:**
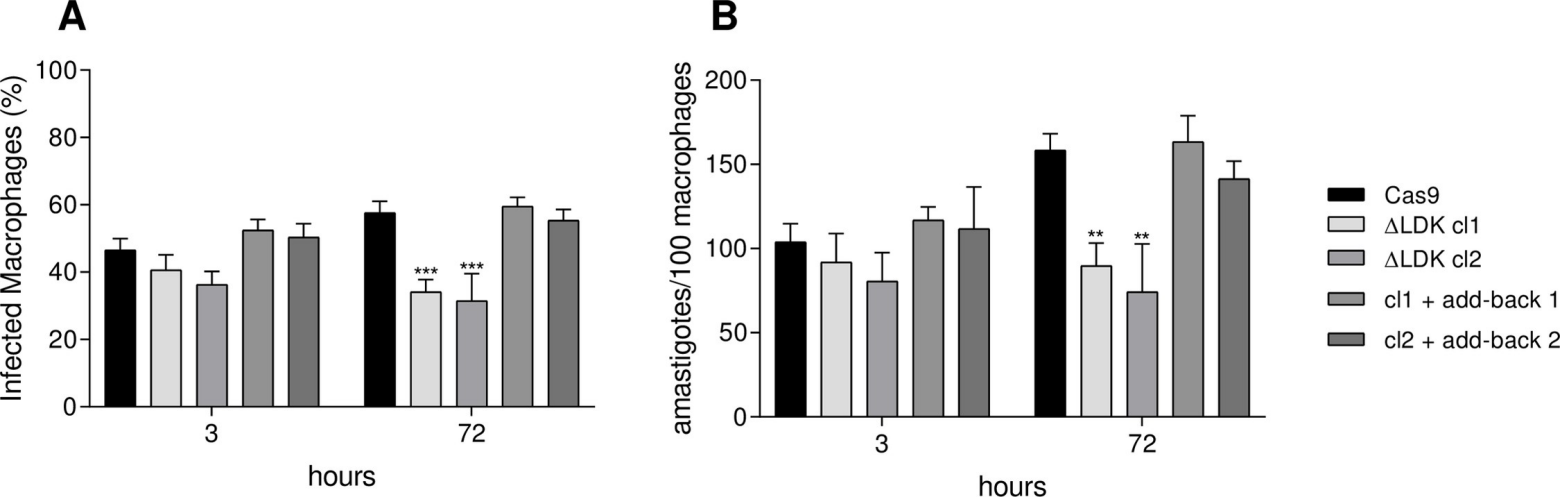
Analysis of the infectivity of Cas9-expressing, ΔLDK null mutants, and add-back parasites in THP-1 macrophages. (A) Percentage of infected macrophages after 3 and 72 h of infection. (B) Number of intracellular amastigotes per 100 macrophages after 3 and 72 h of infection. Ordinary two-way ANOVA test with Dunnett’s post hoc test was used to compare Cas9-expressing, ΔLDK null mutants, and add-back parasites at each time point. *represents significant differences in relation to the Cas9-expressing control (** *p* < 0.01; *** *p* < 0.001).

We investigated the effect of *LDK* deletion in *L*. *infantum* on the infection profile and intracellular parasite proliferation in THP-1 macrophages. After 3 h of infection, there was no difference in the percentage of infected macrophages nor in the number of intracellular amastigotes. However, after 72 h of infection, we verified that *LDK*-knockout parasites had a reduced capacity to infect macrophages and a reduced number of intracellular amastigotes compared to the Cas9-expressing controls (Fig 4A and 4B, respectively). Reintroduction of the *LDK* gene into the *LDK*-knockout clones recovered the phenotype (Fig 4A and 4B).

### *LDK* deletion increased resistance to Sb^III^, but did not change the susceptibility to amphotericin B, miltefosine, and menadione

**Fig 5:**
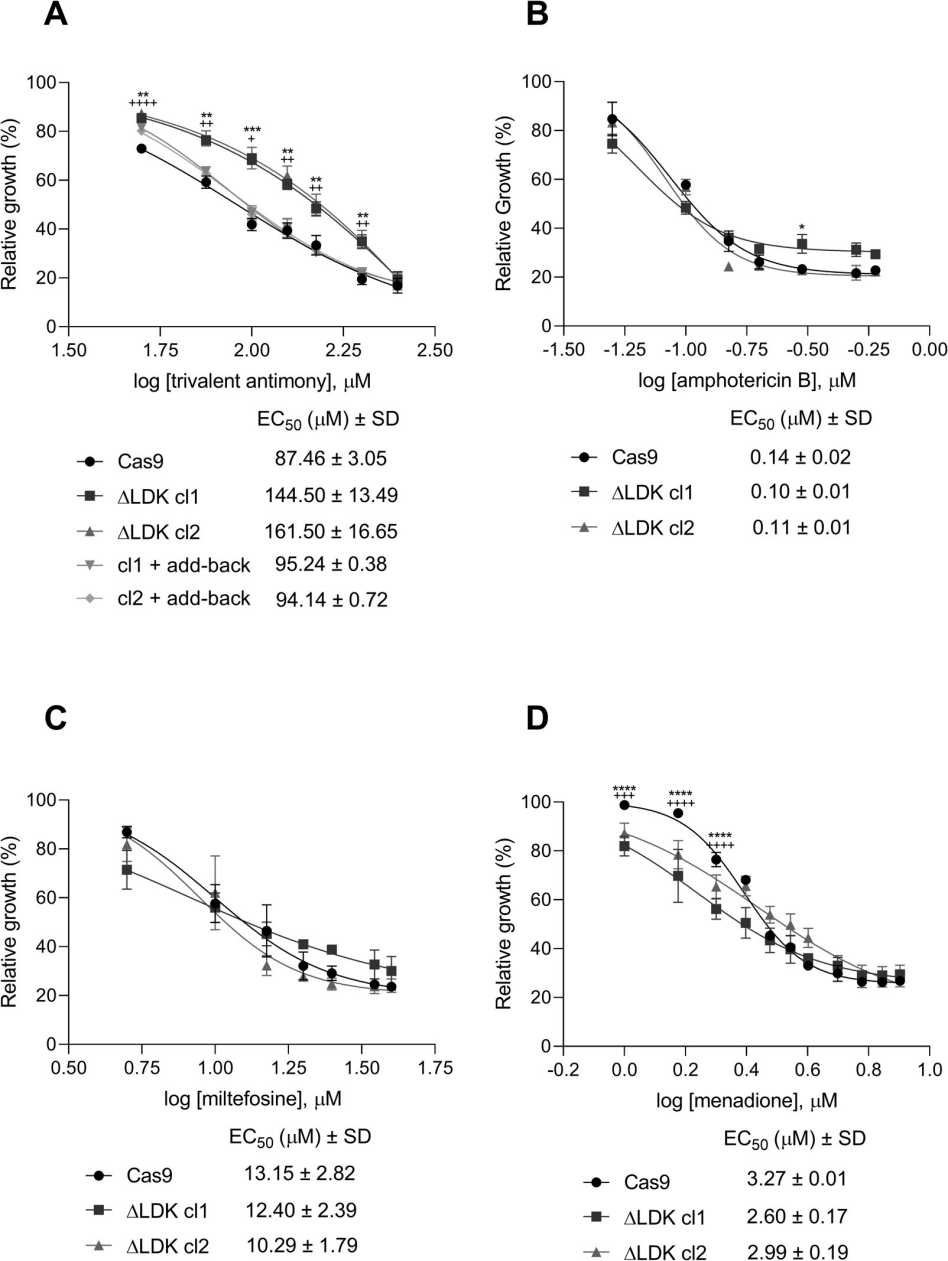
*In vitro* susceptibility assessment of Cas9-expressing, *LDK* knockout, and add-back parasites. Parasites were cultured in the presence of different concentrations of (A) potassium antimony tartrate (Sb^III^) (50 to 250 μM), (B) amphotericin B (0.05 to 0.6 μM), (C) miltefosine (5 to 40 μM), and (D) menadione (1.0 to 8 μM). Parasite growth was determined after 48 h of incubation in the presence or absence of drugs. Data were plotted on dose-response curves in which the mean with standard deviations (SD) of three independent experiments performed in triplicate are represented. The EC_50_ was determined using the linear interpolation method. Two-way ANOVA with post hoc Dunnett’s test was applied to compare Cas9-expressing, *LDK*-knockouts, and add-back parasites for each drug concentration. Differences between Cas9-expressing controls and ΔLDK cl1 (* *p <* 0.05, ** *p* < 0.01; *** *p* < 0.001; **** *p* ≤ 0.0001) and differences between Cas9-expressing controls and ΔLDK cl2 (^+^ *p <* 0.05, ^++^ *p <* 0.01, ^+++^ *p* <0.001; ^++++^ *p* ≤ 0.0001).

We evaluated the susceptibility of both Cas9 and *LDK*-knockout parasites to drugs currently used for leishmaniasis treatment, including antimony, amphotericin B, and miltefosine (Fig 5A–5C). Additionally, we investigated the impact of menadione [21], a compound that induces oxidative stress by increasing peroxide and superoxide radical levels (Fig 5D). Deletion of *LDK* resulted in increased resistance of *L*. *infantum* to Sb^III^ (EC_50_: ΔLDK cl1: 144.50 μM and ΔLDK cl2: 161.50 μM), representing a 1.7 and 1.8 -fold increase in resistance for ΔLDK cl1 and ΔLDK cl2, respectively, compared to the Cas9-expressing control (EC_50_: 87.46 μM). Notably, the reintroduction of the *LDK* gene in the *LDK*-knockout clones restored susceptibility to Sb^III^, as the EC_50_ for add-back parasites (cl1 + add-back: 95.24 μM and cl2 + add-back: 94.14 μM) was similar to that of the Cas9-expressing control (Fig 5A). However, the deletion of LDK did not affect the susceptibility of the parasites to amphotericin B, with EC_50_ values ranging from 0.10 to 0.14 μM for both the Cas9-expressing control and ΔLDK mutant clones (Fig 5B). Similarly, miltefosine susceptibility remained unaltered by LDK deletion, as EC_50_ values for both the Cas9-expressing controls and ΔLDK mutant clones ranged from 10.29 to 13.15 μM (Fig 5C). Additionally, *LDK*-knockout parasites exhibited similar tolerance to oxidative stress induced by menadione (EC_50_: ΔLDK cl1: 2.60 μM and ΔLDK cl2: 2.99 μM) compared to the Cas9-expressing control (EC_50_: Cas9: 3.27 μM) (Fig 5D). No statistically significant differences were observed between the Cas9-expressing control and *LDK*-knockout *L*. *infantum* at any tested concentrations of amphotericin B, miltefosine, and menadione.

## Discussion

Lipid droplets (LDs) are organelles with critical roles in various cellular processes, including lipid metabolism, energy homeostasis, cell signaling, membrane trafficking, inflammation, and innate immunity [10,11,22]. The formation, composition, and functions of LDs are tightly regulated and can vary depending on stimuli, cell types, and the inflammatory environment [11]. LDs contain numerous associated proteins [7,11,22], and in trypanosomatids, two enzymes, TbLpn [12] and LDK [6], have been identified as involved in LD production. In this study, we provide the first evidence of the impact of *LDK* gene deletion using CRISPR/Cas9 on the phenotype of *L*. *infantum*.

We successfully generated *LDK*-null mutants through CRISPR/Cas9 technology and reintroduced the *LDK* gene into add-back parasites, resulting in high levels of *LDK* transcripts in these parasites. Trypanosomatids have unique molecular characteristics, such as polycistronic transcription, mRNA maturation via trans-splicing, and post-transcriptional regulation of gene expression [23]. The untranslated regions (UTRs) play a crucial role in gene expression by influencing mRNA processing, translation, and degradation [24,25]. Our study employed exogenous UTRs flanking the *LDK* gene in add-back parasites. However, it remains uncertain whether the increased mRNA transcript levels lead to elevated protein levels due to post-transcriptional regulation. Our phenotypic assessment indicated strikingly similar results between add-back and Cas9 parasites, suggesting no substantial difference in LDK protein levels between these groups.

Our data indicate that LDK is not essential for *L*. *infantum*, as *LDK*-knockout parasites exhibited no alterations in growth patterns, mirroring the behavior of Cas9-expressing controls. These results align with a previous study in which a reduction in *LDK* expression via RNA interference in *T*. *brucei* did not impact the growth rate [6].

We employed flow cytometry, using both Nile red and Bodipy 493/503, to quantify LDs and lipid levels, as this technique has been utilized in various cell types [26–30]. In trypanosomatids, it has also been employed to quantify LDs [31,32]. Our study successfully employed these methodologies to quantify LDs.

LDs are associated with virulence in several infectious diseases, including *Toxoplasma gondii* and *Plasmodium falciparum* [33]. Similarly, LDs have been linked to virulence in *L*. *infantum*, with their numbers increasing during metacyclogenesis and in intracellular amastigotes [34]. In our study, *LDK* deletion in *L*. *infantum* reduced the amount of LDs during the stationary phase, although complete depletion did not occur. This finding is consistent with previous research that reported reduced LD abundance when *LDK* expression was diminished in *T*. *brucei* [6], suggesting that LDK is essential for maintaining normal LD levels in *L*. *infantum*.

Cells tend to synthesize more LDs in response to increased fatty acids in the medium or lipid homeostasis imbalances [6]. Therefore, we investigated whether *LDK*-knockout parasites would still respond to stimuli that typically induce LD biogenesis. We treated parasites with myriocin, a specific inhibitor of the serine palmitoyltransferase enzyme, a key player in sphingolipid metabolism [18,35], known to induce LD production [18]. Our results demonstrated that treatment with 1.5 μM myriocin for 24 h increased LD production in both Cas9-expressing control and *LDK*-knockout clones, compared to untreated parasites. Importantly, *LDK*-knockout clones exhibited a considerable reduction in LD amount in both growth phases compared to the Cas9-expressing controls in the presence of myriocin. This suggests that LDK deletion may cause changes in other enzymes or pathways indirectly involved in LD biogenesis.

Interestingly, the quantity of LDs in protozoan parasites has been linked to their virulence. In *L*. *infantum*, LDs play a significant role in infection, as they are responsible for producing prostaglandins like prostaglandin F2α, which is associated with infection and the enhanced survival of parasites within macrophages [34]. Our study indicated that LDK deletion in *L*. *infantum* resulted in a reduced number of LDs during the stationary phase. Consequently, we examined its impact on THP-1 macrophage infection and observed that *LDK*-knockout parasites exhibited a decreased ability to maintain infection in macrophages, with a lower intracellular multiplication rate compared to control parasites 72 h post-infection.

Furthermore, we found that *LDK*-knockout increased parasite resistance to Sb^III^. Lipidomic studies have demonstrated that resistance to antimonials in *Leishmania* is linked to altered lipid composition, including increased levels of very-long-chain fatty acids and ergosterol, suggesting modifications in membrane composition in resistant parasites [36,37]. Our results suggest that LDK deletion may alter the metabolism of other lipids, contributing to the observed resistant phenotype. No significant changes were observed in the susceptibility of *LDK*-knockout parasites to amphotericin B and miltefosine. Resistance of *Leishmania* promastigotes to amphotericin B is characterized by alterations in membrane composition and fluidity, along with changes in the machinery for scavenging reactive oxygen species [38,39]. Although miltefosine is believed to affect lipid metabolism, miltefosine-resistant *Leishmania* parasites did not exhibit significant changes in lipid profiles, suggesting that miltefosine resistance could be attributed to distinct biological processes unrelated to lipid biosynthesis [36]. Additionally, LDK deletion did not alter the mechanisms involved in oxidative stress, as there was no difference in parasite viability after treatment with menadione, a compound that generates oxidative stress by increasing peroxide and superoxide radical levels [21].

In conclusion, our study demonstrated that LDK deletion reduces LD production in *L*. *infantum* during the stationary phase. Although treatment with myriocin induced LD production, *LDK*-knockout parasites exhibited reduced LD amount in both the logarithmic and stationary growth phases compared to control parasites. *LDK*-knockout mutants showed a decreased ability to maintain infection in macrophages and were more resistant to Sb^III^, suggesting that this deletion may promote changes in pathways related to Sb^III^ metabolism. The biogenesis of lipid droplets in trypanosomatids, notably in *Leishmania*, is not fully characterized, and is a process that suggests the involvement of several enzymes, related to different pathways. These findings suggest that LDK is crucial for maintaining normal LD levels in *L*. *infantum*.

## Supporting information

S1 Table List of primers used in this study. (DOCX)

S1 Fig Growth curve of promastigote forms of ΔLDK mutant clones. An initial inoculum of 1 x 10^6^ parasites per mL was prepared for the Cas9 parasites, ΔLDK mutant clones cl1 and cl2 and add-back parasites, which were counted every 24 h using the Z1 Coulter Counter. Data represent the mean of three independent experiments performed in triplicates.
(TIF)

S2 Fig Quantification of lipid droplets of Cas9, *LDK*-knockouts, and add-back parasites. (A) The graphs represent the mean geometric fluorescence intensity (gMFI) of Bodipy 493/503. (B) Representative histograms of gMFI of the Bodipy 493/503. (C) Representative images of lipid droplets obtained by confocal microscopy. Two-way ANOVA with Dunnett’s post hoc test was applied to compare Cas9-expressing controls and *LDK*-knockouts for each growth phase. *represents significant differences between Cas9 and ΔLDK cl1 and ΔLDK cl2 knockouts (** *p* < 0.01). Bars: 10 μm.
(TIF)
