# Supplementary material for: Deletion of the lipid droplet protein kinase gene affects lipid droplets biogenesis, parasite infectivity, and resistance to trivalent antimony in Leishmania infantum
Source: PLoS Negl Trop Dis. 2024 Jan 18;18(1):e0011880. doi: 10.1371/journal.pntd.0011880 (PMC10795987; doi:10.1371/journal.pntd.0011880)
Supplement: S1 Table — (DOCX) [file pntd.0011880.s001.docx]

**Supporting information**

**S1 Table. List of primers used in this study.**

| **PRIMER NAME** | **PRIMER SEQUENCE 5’ → 3’** |
| --- | --- |
| LiLDKup_donor_FW | CACAGTCTTTGCGCTGGACGGCTAAACTGAgtataatgcagacctgctgc |
| LiLDKdw_donor_RV | TCAAAGTAGCGTGGCGCATGCGTACATAAGccaatttgagagacctgtgc |
| LiLDK5’sgRNA_FW | gaaattaatacgactcactataggAACGAAAGGCACTGACGAGAgttttagagctagaaatagc |
| LiLDK3’sgRNA_FW | gaaattaatacgactcactataggTATGCGTGCGTTTGCCACGAgttttagagctagaaatagc |
| sgRNA_RV | aaaagcaccgactcggtgccactttttcaagttgataacggactagccttattttaacttgctatttctagctctaaaac |
| Li5’LDK_FW (P1) | GCTGGACGGCTAAACTGAAA |
| BLAST.mid_RV (P2) | CCATCACTGTCCTTCACTATCG |
| NEO.mid_RV (P3) | GCCAACGCTATGTCCTGATA |
| LiLDKcds_FW (P4) | ATGACCACAAGGAAAGTGATCGG |
| LiLDKcds_RV (P5) | TCACGCATGGCCCGTCTTA |
| RTqPCR_LiLDK_FW | TACGATCGAGTGCGGTACTA |
| RTqPCR_LiLDK_RV | CCCAAAGTCAGAGAGCAACA |

RTqPCR_DNApol_FW CGAGGGCAAGACATAC

RTqPCR_DNApol_RV GAGAGCGGGCACCAATCAC
