# Supplementary figures and images for: Deletion of the lipid droplet protein kinase gene affects lipid droplets biogenesis, parasite infectivity, and resistance to trivalent antimony in Leishmania infantum
Source: PLoS Negl Trop Dis. 2024 Jan 18;18(1):e0011880. doi: 10.1371/journal.pntd.0011880 (PMC10795987; doi:10.1371/journal.pntd.0011880)

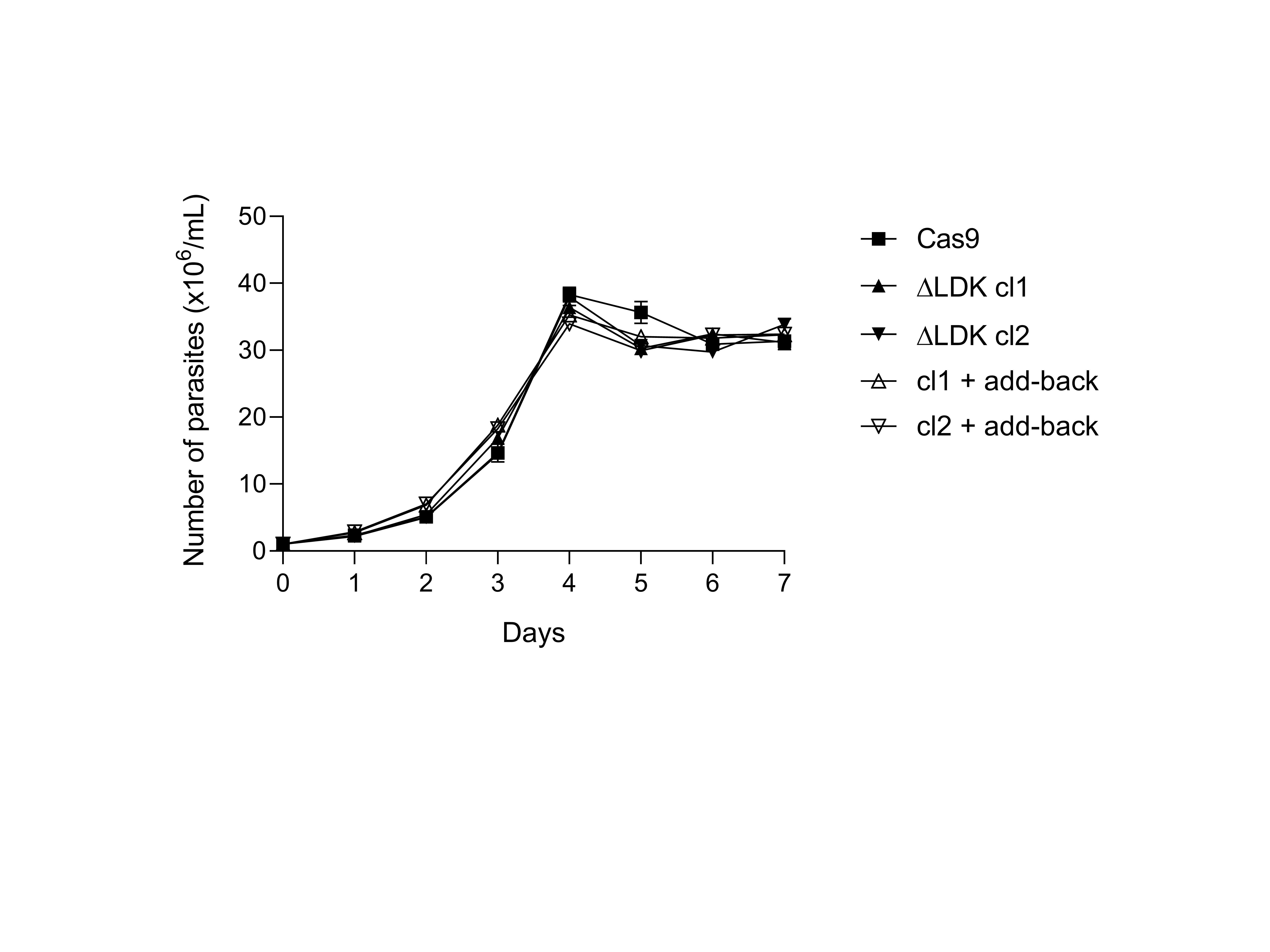

Supplement: S1 Fig — An initial inoculum of 1 x 10^6^ parasites per mL was prepared for the Cas9 parasites, ΔLDK mutant clones cl1 and cl2 and add-back parasites, which were counted every 24 h using the Z1 Coulter Counter. Data represent the mean of three independent experiments performed in triplicates. (TIF) [file pntd.0011880.s002.tif]

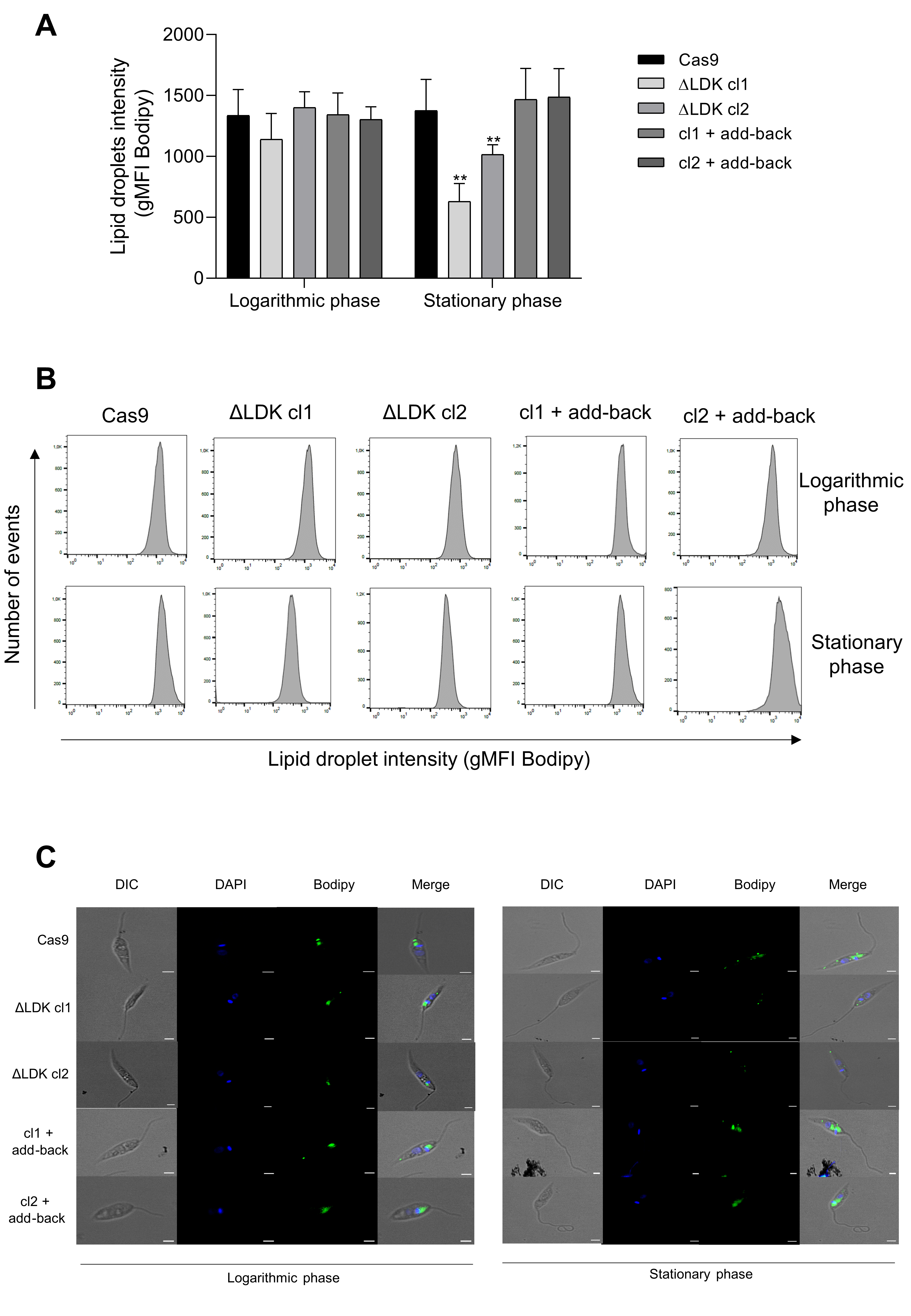

Supplement: S2 Fig — (A) The graphs represent the mean geometric fluorescence intensity (gMFI) of Bodipy 493/503. (B) Representative histograms of gMFI of the Bodipy 493/503. (C) Representative images of lipid droplets obtained by confocal microscopy. Two-way ANOVA with Dunnett’s post hoc test was applied to compare Cas9-expressing controls and LDK-knockouts for each growth phase. *represents significant differences between Cas9 and ΔLDK cl1 and ΔLDK cl2 knockouts (** p < 0.01). Bars: 10 μm. (TIF) [file pntd.0011880.s003.tif]
